# Tracking the polyclonal neutralizing antibody response to a dengue virus serotype 1 type-specific epitope across two populations in Asia and the Americas

**DOI:** 10.1038/s41598-019-52511-z

**Published:** 2019-11-07

**Authors:** Daniela V. Andrade, Colin Warnes, Ellen Young, Leah C. Katzelnick, Angel Balmaseda, Aravinda M. de Silva, Ralph S. Baric, Eva Harris

**Affiliations:** 1 Division of Infectious Diseases and Vaccinology, School of Public Health, University of California, Berkeley, Berkeley, CA USA; 2 Department of Epidemiology, Gillings School of Public Health, University of North Carolina, Chapel Hill, CA USA; 3 National Virology Laboratory, National Center for Diagnosis and Reference, Ministry of Health, Managua, Nicaragua; 4 Department of Microbiology and Immunology, School of Medicine, University of North Carolina, Chapel Hill, NC USA

**Keywords:** Viral infection, Dengue virus

## Abstract

The four dengue virus serotypes (DENV1-4) cause major public health problems worldwide. Highly neutralizing type-specific human monoclonal antibodies (hmAbs) target conformation-dependent epitopes on the DENV envelope protein, including 1F4, a DENV1 type-specific hmAb. Using a recombinant DENV2 virus displaying the DENV1 1F4 epitope (rDENV2/1), we measured the proportion and kinetics of DENV1 neutralizing antibodies targeting the 1F4 epitope in individuals living in Asia and the Americas where different DENV1 genotypes were circulating. Samples from 20 individuals were analyzed 3 and 18 months post-primary DENV1 infection, alongside samples from 4 individuals collected annually for four years post-primary DENV1 infection, from two studies in Nicaragua. We also analyzed convalescent post-primary DENV1 plasma samples from Sri Lankan individuals. We found that neutralizing antibodies recognizing the 1F4 epitope vary in prevalence across both populations and were detected from 20 days to four years post-infection. Additionally, both populations displayed substantial variability, with a range of high to low proportions of DENV1 type-specific neutralizing antibodies recognizing the 1F4 epitope seen across individuals. Thus, the 1F4 epitope is a major but not exclusive target of type-specific neutralizing antibodies post-primary infection with different DENV1 genotypes in Asia and Latin America, and additional epitopes likely contribute to type-specific neutralization of DENV1.

## Introduction

The four dengue virus serotypes (DENV1-DENV4) are positive-sense RNA arboviruses in the family *Flaviviridae*. Widely distributed across many geographical regions, DENV causes the most prevalent human mosquito-borne viral disease worldwide, with over one third of the world’s population at risk of infection^1,2^. Although asymptomatic infection occurs frequently, infection with DENV may cause a spectrum of disease manifestations, ranging from classical dengue fever (DF) to more severe dengue hemorrhagic fever (DHF) and dengue shock syndrome (DSS)^3–5^. While severe manifestations may occasionally be reported during primary infection, a second infection with a heterologous DENV serotype is the main risk factor for DHF/DSS^6^.

Neutralizing antibodies are a correlate of protection against symptomatic DENV infection^7–9^. After a first natural infection with any of the four DENV serotypes, type-specific neutralizing antibodies directed to the homologous serotype are elicited. In addition to these type-specific antibodies, which are thought to be life-long, a large population of cross-reactive antibodies is generated, which in turn may be protective or may enhance infection and disease with heterologous serotypes^10–12^. Given this risk, it is imperative that vaccines elicit balanced and protective immunity to all four DENV serotypes simultaneously.

The envelope (E) glycoprotein is the major protein on the surface of the virion, where it forms parallel head-to-tail homodimers that lie in rafts on the surface of mature viruses^13–15^. Across DENV serotypes, the amino acid homology of the E protein is 60–70%^16^. The E protein ectodomain is the major target of neutralizing antibodies and consists of three domains: EDI, EDII and EDIII^13,17–19^. EDI is formed into an eight-stranded β-barrel and comprises the central region of the E monomer. EDII contains a highly conserved fusion loop that facilitates fusion of the virus with endosomal membranes under mildly acidic conditions^20^. EDIII has an immunoglobulin-like fold and is involved in receptor recognition^21^.

Isolation of human monoclonal antibodies (hmAbs) from individuals with a history of DENV infection has been fundamental for elucidating the specificity and mechanisms of the neutralizing antibody response to DENV infection. One of the key findings demonstrated that potent type-specific hmAbs target epitopes that require the intact E protein to be assembled on the DENV virion^22–24^. One such potent type-specific antibody is 1F4, a DENV1 type-specific hmAb^22,25^. However, unlike a number of hmAbs that recognize quaternary epitopes, 1F4 does not bind across neighboring E proteins. Instead, the footprint of 1F4 is located within an E monomer and spans mostly across EDI, although interaction on the EDI/EDII hinge region is also described^25^. The conformation of the E protein adopted in the context of the virion is essential for binding to the 1F4 hmAb^25^. A second DENV1 type-specific and strongly neutralizing hmAb, 14c10, has an epitope that overlaps with the 1F4 epitope^25^. The 14c10 epitope is quaternary in nature because it spans EDI and EDIII of different E molecules on the surface of the virus^26^.

Reverse genetics approaches have been employed to transplant the core of quaternary epitopes targeted by DENV type-specific hmAbs into a distinct DENV backbone to create chimeric viruses^27–29^. We have previously shown that primary DENV3 polyclonal sera of a large number of individuals from a dengue-endemic area track to varying degrees with the DENV3 type-specific 5J7 epitope transplanted into a DENV4 backbone^30,31^. The DENV2-specific neutralizing antibody response was shown to track with the 2D22 quaternary epitope in samples from individuals who experienced natural DENV infection^32^. Similarly, chimeric viruses have been successfully used to track the antibody response of individuals who were immunized with a monovalent live attenuated DENV2 vaccine^29^ or tetravalent dengue vaccine candidates^33,34^. All together, these studies support the pertinence of chimeric dengue viruses as tools for dissecting the specificity of the polyclonal neutralizing antibody response and helping validate the footprint of novel epitopes.

We have designed and recovered a recombinant DENV2 virus with a transplant of the DENV1 1F4 conformational epitope. This chimeric virus has been validated for structural integrity and used to map DENV1 type-specific antibodies stimulated by vaccination^35^. Here, we use the DENV2/1 virus to quantify the proportion and kinetics of neutralizing antibodies directed to this epitope in individuals who experienced primary DENV1 infection. Of note, we analyzed individuals in two dengue-endemic regions where different genotypes of DENV1 are circulating – in Asia and in the Americas – at various time-points post-infection, which informs about the prevalence and durability of this epitope in light of genotypic variations within the DENV1 serotype. Our results show that 1F4-specific neutralizing antibody is prevalent in individuals in both endemic areas, and from ~20 days to as late as four years post-infection. Importantly, the proportion of the DENV1 type-specific response to this target showed substantial variability from high to low recognition of the 1F4 epitope across the study population, which suggests that the DENV1 repertoire is comprised of more than one immunodominant epitope. In sum, our findings validate the 1F4 epitope as an important component of the DENV1 type-specific neutralizing response in two dengue-endemic regions globally. The identification of the determinants of protection against the DENV1 serotype across a large number of individuals has implications for design and evaluation of dengue vaccines. Moreover, these results reiterate the relevance of further studies aimed at characterizing other antigenic sites on DENV1.

## Results

### Study participants

Twenty patients enrolled in the Nicaraguan Dengue Hospital-based Study were selected for analysis of the proportion and kinetics of the DENV1 response directed to the 1F4 epitope. We used plasma samples collected 3 and 18 months post-primary DENV1 infection. All cases were confirmed to be positive for DENV1 by reverse-transcription PCR (RT-PCR) and/or virus isolation. All individuals experienced only one primary infection with DENV1 in 2007 or 2012, with 16 manifesting disease as DF and 4 as DHF (Supplementary Table S1). For analysis of the 1F4 response from one to four years post-illness, we selected 4 individuals enrolled in the Pediatric Dengue Cohort Study who experienced one RT-PCR-confirmed infection with the DENV1 serotype in 2005 or 2009 and had four subsequent annual samples without manifesting symptomatic infection or serological evidence of inapparent infection as demonstrated by Inhibition ELISA titers (Supplementary Table S2). All 4 individuals manifested disease as DF. To analyze the response to the 1F4 epitope in a population in Sri Lanka, we selected 12 samples collected post-primary DENV1 infection. The DENV1 genotype V was circulating in Nicaragua, while DENV1 genotype I was circulating in Sri Lanka.

### Polyclonal response post-primary DENV1 infection tracks with a DENV1 type-specific conformational epitope transplanted into a heterologous DENV backbone

**Figure 1:**
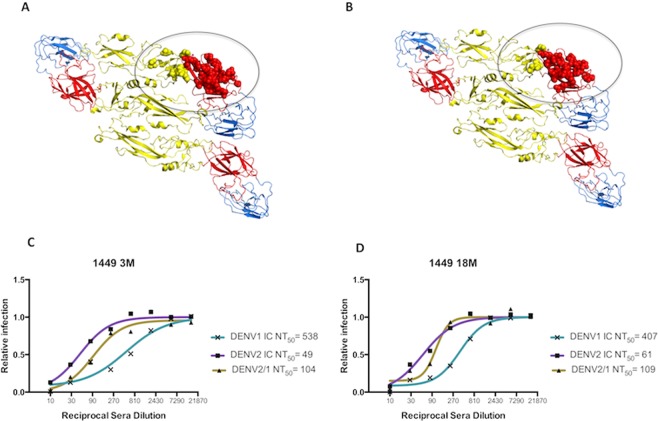
Amino acid residues comprising the 1F4 epitope in the EDI and EDI/EDII hinge region of a DENV1 E protein monomer were transplanted into a DENV2 backbone and analyzed by neutralization assay. (**A**) Diagram of the DENV1 E dimer (domains I, II and III in red, yellow and blue, respectively) with yellow and red spheres representing the 1F4 epitope footprint. (**B**) The amino acid residues within the 1F4 epitope, represented in red and yellow spheres, were transplanted into a DENV2 backbone, creating the rDENV2/1 virus. (**C,D**) Representative sigmoidal dose-response curves used to estimate the 50% neutralization titer (NT_50_) to the rDENV2/1 and parental DENV1 and DENV2 viruses in plasma samples from patient 1449 at 3 (**C**) and 18 months (**D**).

Recent findings reveal that the majority of potent DENV neutralizing hmAbs recognize epitopes that are only present on the assembled virion^23–25,36^. One such hmAb is 1F4, a conformation-dependent DENV1 type-specific hmAb isolated from an individual who experienced primary infection with DENV1^22^. The 1F4 epitope footprint is located on EDI and the EDI/EDII hinge region within an E protein monomer and consists of 26 amino acids, represented by red and yellow spheres in Fig. 1A ^25^. Thirty unique amino acid residues encompassing the core of the DENV1 1F4 epitope were transplanted onto a DENV2 backbone, creating the rDENV2/1 virus (Fig. 1B), which was efficiently neutralized by hmAb 1F4, but not 14c10^35^. To determine whether DENV1 type-specific antibodies target the 1F4 epitope in the polyclonal sera of individuals who experienced a primary infection with DENV1, neutralization assays were performed with plasma samples collected at different time-points post-illness (3 and 18 months) against the rDENV2/1 virus displaying the 1F4 epitope and its parental DENV1 and DENV2 strains. Representative sigmoidal dose-response curves displaying neutralizing antibody titers (NT_50_) of plasma samples from individual 1449 indicate that antibodies in polyclonal sera recognize the 1F4 epitope transplanted into the DENV2 backbone at both 3 and 18 months post-infection (Fig. 1C,D). In addition, the maintenance of rDENV2/1 neutralizing antibody titers despite a decrease in the parental DENV1 NT_50_ values in the later time-point indicates a lasting neutralizing antibody response directed to the 1F4 epitope.

### Neutralizing antibody titers to the rDENV2/1 virus are maintained at 3 and 18 months post-infection

**Figure 2:**
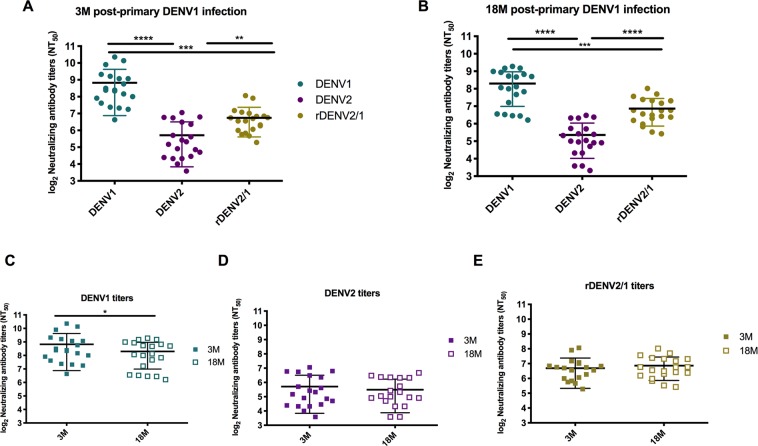
Inclusion of the 1F4 epitope amino acid residues in a DENV2 backbone results in gain of neutralization against rDENV2/1 chimeric virus by polyclonal sera post-primary DENV1 infection. (**A**,**B**) Primary DENV1 plasma from 20 individuals in the Nicaraguan hospital-based study strongly neutralize the parental DENV1 virus and gain neutralization capacity against a DENV2 backbone containing the 1F4 amino acid residues (rDENV2/1) at 3 and 18 months post-infection. The NT_50_ values to the rDENV2/1 virus and the DENV1 and DENV2 parental viruses were compared by one-way ANOVA (n = 20). (**C**–**E**) Paired analysis of the longitudinal samples at 3 and 18 months shows a significant decay of DENV1 NT_50_ values, whereas DENV2 and rDENV2/1 titers are maintained constant over time. The t-test was used to compare the neutralizing antibody titers to each serotype at 3 and 18 months post-infection. Data are representative of two independent experiments, and samples were processed in duplicate for each plasma sample. **p < 0.01; ***p < 0.001; ****p < 0.0001.

The kinetics of the DENV-specific antibody response after a first infection is characterized by the appearance of IgM antibodies, followed by a durable IgG response. The early IgG response contains diverse antibody populations, including cross-neutralizing antibodies and type-specific neutralizing antibodies. While some cross-neutralizing antibodies are transient, type-specific antibodies are believed to be life-long and can be detected years after infection^37^. We used the rDENV2/1 virus to specifically determine how the primary DENV1 antibody response tracks with the 1F4 conformational epitope in multiple individuals. As above, we performed neutralization assays with primary DENV1 plasma against the rDENV2/1 virus and its DENV1 and DENV2 parental viruses. As expected, in all subjects, primary DENV1 plasma strongly neutralized the parental DENV1 virus at both time-points post-infection (Fig. 2A,B). Also as expected, the NT_50_ values to the homologous serotype DENV1 were significantly higher than to the heterologous serotype DENV2 (Fig. 2A,B). At both time-points, the NT_50_ values to rDENV2/1 were significantly higher than the parental DENV2 titers and significantly lower than the parental DENV1 titers. Paired analysis of longitudinal samples at 3 and 18 months revealed significant decay of DENV1 NT_50_ values (Fig. 2C), while DENV2 and rDENV2/1 titers remain constant (Fig. 2D,E). In fact, the low levels of DENV2 cross-neutralizing antibody titers at the earlier time-point could explain the lack of a detectable change at the later time-point. Altogether, this longitudinal analysis suggests that the antibodies targeting the 1F4 epitope are maintained over time despite the decay of DENV1 NT_50_ values, an indication of the durability of this epitope-specific response.

### The proportion of the DENV1 neutralizing response attributable to the 1F4 epitope varies across the Nicaraguan population

**Figure 3:**
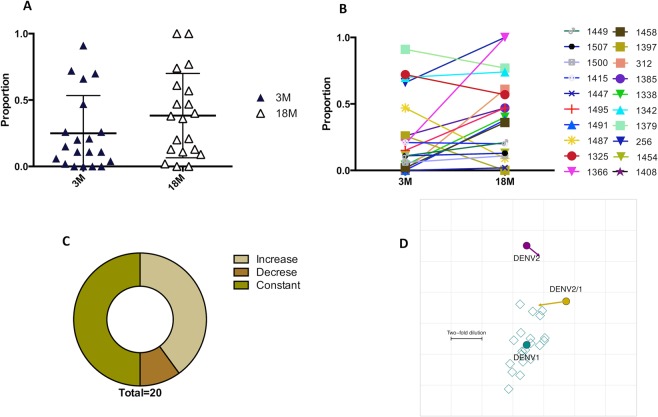
The proportion of the DENV1 type-specific neutralizing antibody response targeted to the 1F4 epitope is highly variable across the Nicaraguan population in the hospital-based study at 3 and 18 months post-infection. (**A**) Analysis of the proportion of the DENV1 type-specific response attributable to the 1F4 epitope at 3 and 18 months reveals substantial variability, with individuals displaying a range from high to low proportions of the DENV1 response directed to the 1F4 epitope. (**B**) Paired analysis where each individual was assigned a color and a symbol to enable visualization of the trajectory of the DENV1 type-specific response to the 1F4 epitope at 3 and 18 months post-infection. (**C**) Distribution of individuals who gained recognition of the 1F4 epitope between 3 and 18 months (beige), lost recognition (brown) or remained constant (green). (**D**) The antigenic cartography map positions viruses (DENV1, DENV2 and rDENV2/1 in teal, purple and yellow, respectively) and plasma (open teal squares), with the distance between each virus and plasma derived from its respective neutralizing antibody titer. Each grid square corresponds to a 2-fold dilution in the NT_50_. From 3 to 18 months post-primary DENV1 infection, the DENV1 and rDENV2/1 titers converge, as indicated by the rDENV2/1 arrow pointing towards DENV1.

Next, we determined how much of the DENV1 type-specific response is directed to the 1F4 conformational epitope. Using the formula (rDENV2/1 NT_50_ – DENV2 NT_50_)/(DENV1 NT_50_ – DENV2 NT_50_), where the cross-reactive (DENV2) NT_50_ has been subtracted from both the DENV2/1 and DENV1 NT_50_ titers, we calculated the proportion of the DENV1 neutralizing antibody response attributable to the 1F4 epitope. Across the 20 individuals enrolled in the hospital-based study in Nicaragua, we observed that the mean proportion of the DENV1 type-specific response targeted to the 1F4 epitope was 24.9% at 3 months post-infection and 38.3% at 18 months post-infection (Fig. 3A). This analysis shows a substantial variation in the degree to which DENV1 NT_50_ titers track with the 1F4 epitope: most individuals contained either high or low proportions to the 1F4 epitope. At 3 months post-primary DENV1 infection, the proportion ranges from 0–91%, while at 18 months it ranges from 0–100% (Fig. 3A). Pairwise analysis showed that most individuals had an increase in response to the 1F4 epitope (8/20) or retained the response to the 1F4 epitope (10/20) (Fig. 3B,C) at 3 and 18 months after infection, which is a strong indication of a long-lived pool of antibodies specific to this epitope. To estimate the antigenic similarity between DENV1 and rDENV2/1, we employed the antigenic cartography method^38^. The neutralizing antibody titers of each plasma sample at 3 and 18 months was treated as a measure of the distance between the plasma and the viruses. We observed that the rDENV2/1 is closer to DENV1 than DENV2 at 3 months post-primary DENV1 infection (Fig. 3D). By 18 months, rDENV2/1 moved even closer to DENV1 (Fig. 3D). This analysis indicates that rDENV2/1 and DENV1 share greater similarity at the later time-point, as the DENV1 serum response becomes increasingly specific to the 1F4 epitope over time.

### Individual variation exists in the DENV1 neutralizing antibody response directed to the 1F4 epitope

**Figure 4:**
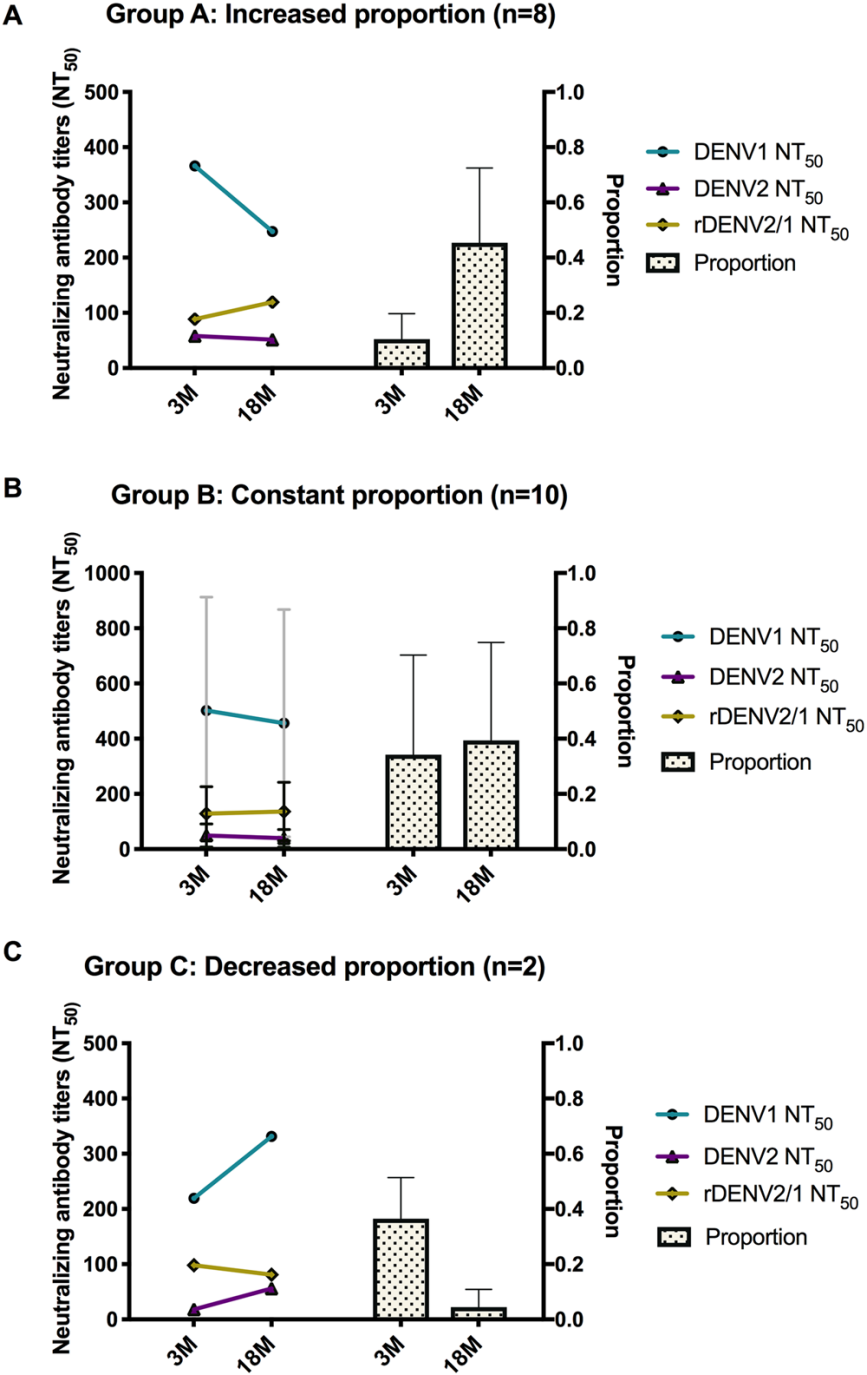
The proportion of the DENV1 neutralizing antibody response directed to the 1F4 epitope is affected by the levels of homotypic and heterotypic titers. (**A**–**C**) To better understand the different patterns of the proportion observed in the individual trajectory analysis, we grouped the individuals who displayed increased (**A**), constant (**B**) or decreased (**C**) proportion over time (right Y-axis) and concomitantly analyzed their NT_50_ values (left Y-axis) to the parental DENV1 (teal line) and DENV2 viruses (purple line) and the rDENV2/1 chimeric virus (yellow line). In group A, the increase in antibodies targeting the 1F4 epitope is associated with a decay in the DENV1 NT_50_ values and concomitant maintenance of the rDENV2/1 titers. In group B, the constant proportion between 3 and 18 months is accompanied by changes in the magnitude of neutralizing antibody titers to the parental viruses, while in Group C, the loss of recognition of the 1F4 epitope is associated with an increase in the NT_50_ values to DENV1 and DENV2 viruses.

To better understand the different patterns illustrated in the individual trajectory analysis in Fig. 3B, we grouped the individuals who displayed increased, constant or decreased proportions of DENV1 neutralizing antibodies attributable to 1F4 over time and concomitantly analyzed their neutralizing antibody titers to the rDENV2/1 virus and the DENV1 and DENV2 parental viruses. As seen in Fig. 4A, individuals who displayed an increase in the 1F4 proportion (Group A) exhibited decay in DENV1 NT_50_ values, while only a slight increase in the rDENV2/1 NT_50_ values was observed. In the subset of individuals who maintained constant proportion over time, we observed the same pattern, although the magnitude of DENV1 NT_50_ values decay was lower than in Group A (Fig. 4B). Finally, the individuals who displayed a decreased 1F4 proportion over time presented a rise in the DENV1 and DENV2 antibody titers (Fig. 4C). In all the groups, the DENV2/1 neutralizing antibody titers remained relatively constant.

### Antibodies directed to the 1F4 epitope can be detected years after primary DENV1 infection

**Figure 5:**
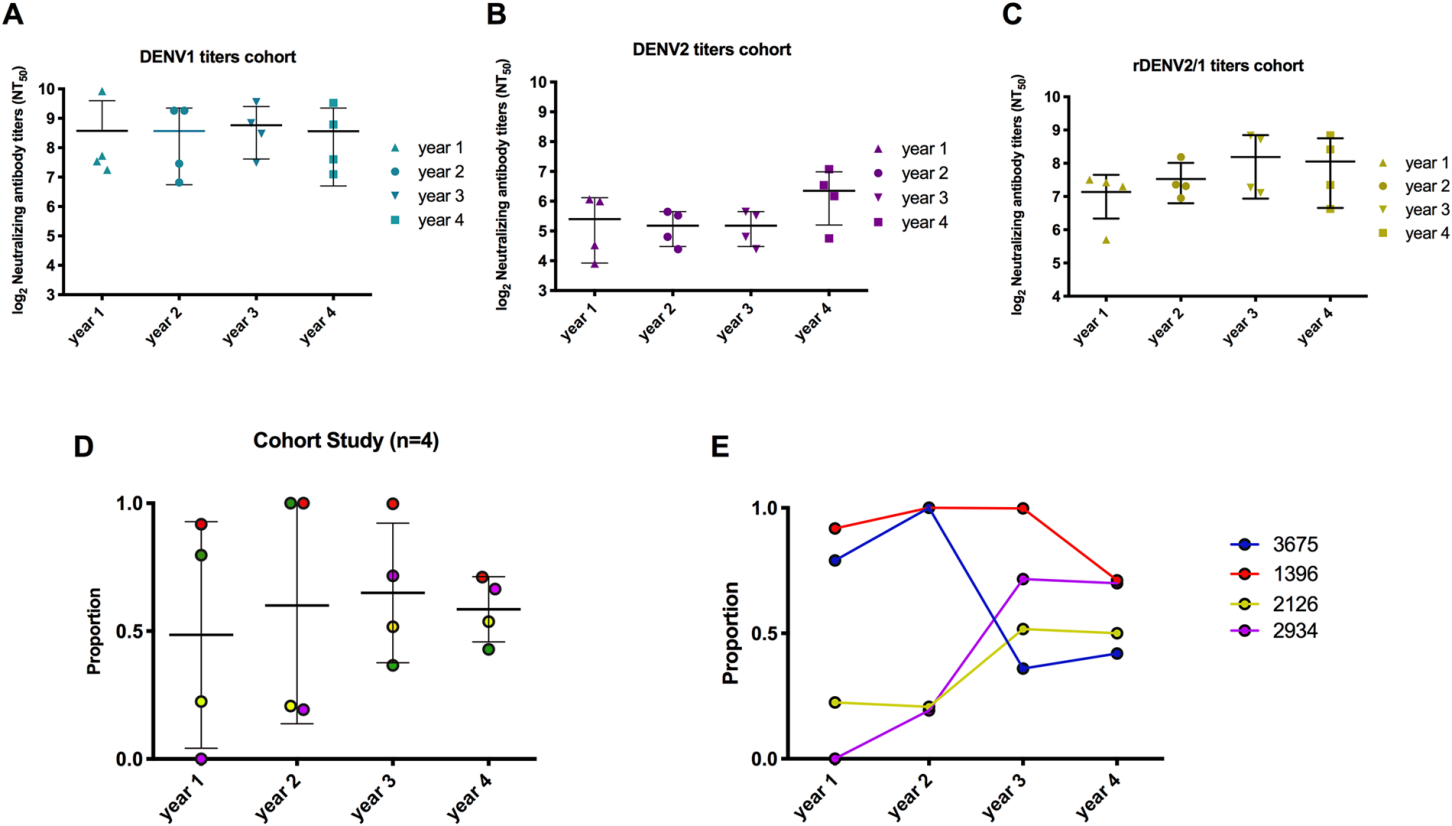
Primary DENV1 plasma samples collected up to four years post-infection track to varying degrees with the 1F4 epitope in the Pediatric Dengue Cohort Study in Nicaragua. (**A**–**C**) The mean of the neutralizing antibody titers to the rDENV2/1 and parental DENV1 and DENV2 viruses did not change significantly over four years post-infection. The NT_50_ values were compared by one-way ANOVA analysis (n = 4). (**D**) From year 1 to year 4 post-primary DENV1 infection, the 1F4 epitope accounts for 47, 48, 52 and 47% of the DENV1 type-specific response, respectively. The proportions were compared by one-way ANOVA analysis (n = 4).

While we demonstrated that DENV1 type-specific antibodies track with the 1F4 epitope in a substantial number of Nicaraguan individuals out to 18 months post-infection, we wanted to evaluate whether this response is maintained over an extended period of time. To test the long-term durability of the DENV1 response directed to the 1F4 epitope, we analyzed samples collected annually 1 to 4 years post-primary DENV1 infection in a long-standing dengue cohort study in Nicaragua. Overall, the average NT_50_ value to the rDENV2/1 virus and both of the parental viruses was maintained from year 1 to year 4 (Fig. 5A–C). With regard to the analysis of the proportion of 1F4, we observed that from year 1 through year 4, the 1F4 epitope accounted on average for 48–65% of the DENV1 neutralizing response (Fig. 5D). However, similar to the population enrolled in the Nicaraguan hospital study, we observed substantial variability among individuals in the amount of the DENV1 type-specific response directed to this conformational epitope over time (Fig. 5D).

### Antibodies to the 1F4 epitope are prevalent in a dengue-endemic area in Asia

The traditional classification of DENV into four genetically distinct serotypes appears to underestimate the impact of viral genotypic variation on neutralizing antibody response^38^. Recent data point to a clear effect of DENV genetic variation among genotypes on neutralization capacity^39,40^. Given the efforts to design a vaccine that is effective for populations exposed to DENV strains of different genotypes, we evaluated whether the polyclonal response in individuals living in a dengue-endemic area where a distinct genotype of DENV1 (genotype I) was circulating also tracks with the 1F4 epitope. The alignment of Nicaragua DENV1 (genotype V), Sri Lanka DENV1 (genotype I) and West Pac DENV1 (genotype IV) highlights three amino acid residue changes in the 1F4 epitope (T → S at position 155 in the Sri Lanka DENV1; T → I at position 161 in the Nicaragua DENV1; S → T at position 171 in the Sri Lanka DENV1) (Supplementary Fig. S1).

**Figure 6:**
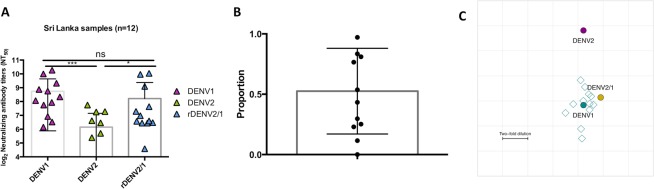
The 1F4 epitope is recognized by polyclonal sera from Sri Lankan individuals in the convalescent phase post-primary DENV1 infection. (**A**) In the convalescent phase, the NT_50_ values to the DENV1 infecting serotype and the rDENV2/1 are significantly higher than the NT_50_ values to the DENV2 serotype in Sri Lankan DENV-infected individuals. (**B**) The proportion of the DENV1 type-specific response attributable to the 1F4 epitope is variable and averages 53% across 12 Sri Lankan individuals. (**C**) The antigenic cartography map positions viruses (DENV1, DENV2 and rDENV2/1 in teal, purple and yellow, respectively) and plasma (12 open teal squares) and indicates a close proximity between DENV1 and rDENV2/1 viruses. Each grid square corresponds to a 2-fold dilution in the neutralization titer. The NT_50_ values were compared by one-way ANOVA analysis (n = 12). *p < 0.05, ***p < 0.001.

From studies in Sri Lanka, we selected 12 individuals who experienced laboratory-confirmed primary DENV1 infection, from whom convalescent phase blood samples were available. We performed a similar analysis as above and found that the highest titers were found to the DENV1 homologous serotype, while cross-neutralizing titers to the heterologous DENV2 serotype were significantly lower (Fig. 6A). While the NT_50_ values to the rDENV2/1 virus varied across the individuals, the average was comparable to the DENV1 NT_50_ values but significantly higher than the DENV2 NT_50_ values (Fig. 6A). On average, 52% of the DENV1 neutralizing antibody response targeted the 1F4 epitope across the 12 Sri Lankan individuals (Fig. 6B), higher than in the Nicaraguan samples. Similar to the Nicaraguan population, we also observed a cluster of individuals with a high proportion, as well as a cluster of individuals with a low proportion of the 1F4 epitope (Fig. 6B). Finally, the antigenic cartography map again supports the antigenic similarity between rDENV2/1 and DENV1, as indicated by the close distance between these viruses and the primary DENV1 sera (Fig. 6C). Notably, the parental DENV1 West Pac 74 is in genotype IV, which is more closely related to genotype I circulating in Sri Lanka than to genotype V circulating in Nicaragua^41–43^.

## Discussion

While the overall homology of the E protein is 60–70% across serotypes, the type-specific epitopes targeted by hmAbs appear to be located in distinct regions of the E protein on the virion. As shown by structural studies, the footprint of the DENV3 type-specific hmAb 5J7 is located around the EDI/EDII hinge region, and it spans across three different molecules within a single raft^24^. The DENV2 type-specific hmAb 2D22, on the other hand, targets mostly EDIII and EDII including the fusion loop^23,29^, whereas the DENV1 type-specific 1F4 epitope is centered around EDI and extends to the EDI/EDII hinge region^25^. Epitope mapping of escape mutants evidenced two independent single-nucleotide mutations in the 1F4 epitope (amino acid changes at positions 274 in the DI-DII hinge and 47 in DI of the E protein) that resulted in loss of neutralization^22^. As recent studies highlight, polyclonal sera of naturally DENV-infected individuals or recipients of dengue vaccines track to varying degrees with complex quaternary epitopes of the 5J7 and 2D22 hmAbs^31,32^. To date, two potent DENV1 type-specific mAbs have been isolated and characterized, namely 1F4^25^ and 14c10^26^. Sera from recipients of the Dengvaxia® tetravalent dengue vaccine were shown to track with the 1F4 epitope transplanted into a DENV3 backbone^33^. Here, we employ a chimeric virus, rDENV2/1, displaying the 1F4 epitope to measure the prevalence of antibodies targeting this epitope across two populations in Asia and in the Americas. Of note, this is the first time chimeric viruses have been applied to measure a type-specific epitope in areas affected by distinct genotypes, hence providing a deeper analysis of the DENV1 epitope repertoire.

Results of the Phase IIb clinical trial of the Dengvaxia vaccine demonstrated reduced protection against DENV2 disease, despite high seroconversion rates and geometric mean neutralization titers to DENV2^44^. One plausible explanation is that the vaccine did not induce DENV2 type-specific neutralizing antibodies but instead, a large population of cross-reactive neutralizing antibodies^34^. In this context, tools that enable measurement of the type-specific neutralizing antibody response elicited by vaccination are needed for guiding vaccine design and evaluation. While it has been shown that type-specific antibodies drive most of the neutralization in post-primary sera^34,45,46^, the viral epitopes targeted by these antibodies in a larger population remain to be examined. Therefore, approaches that dissect the specificity of the polyclonal antibody response elicited by natural infection are relevant and needed. In our longitudinal analysis of the neutralizing antibody titers at 3 and 18 months post-primary DENV1 infection, we observed low levels of neutralization of the DENV2 heterologous serotype. These plasma samples gained neutralization capacity when amino acid residues of the DENV1 type-specific 1F4 epitope were transplanted into a DENV2 backbone, indicating that this epitope is specifically recognized by a DENV1 type-specific antibody population in polyclonal sera post-primary DENV1 infection.

In the Nicaraguan population, the proportion of the DENV1 neutralizing antibody response attributable to the 1F4 epitope varied substantially across individuals, with some individuals displaying very high levels of antibodies targeting the 1F4 epitope, another group with nonexistent or very low levels of the DENV1 antibody response directed to this conformational epitope, and others in the middle. Such findings suggest the existence of other immunodominant epitopes within the DENV1 repertoire, such as the hmAb 14c10^26^. While the 1F4 footprint is confined to EDI and the EDI/II hinge region of a single E molecule, the footprint of 14c10 includes the EDI/II hinge region and EDIII of adjacent E proteins on the viral surface^26^. Structural studies revealed that both 14c10 and 1F4 hmAbs bind to overlapping regions on the EDI/EDII hinge, which could indicate that type-specific neutralizing antibodies preferentially target this region on the E protein. Further studies are needed to assess the importance of 14c10 and other epitopes as targets of human type-specific DENV1 neutralizing antibodies. Furthermore, intrinsic host genetic polymorphisms may affect the germline-encoded antibodies and subsequent antigen recognition, which could then partially explain the variability in 1F4 recognition across the study population.

Besides the heterogeneous prevalence of neutralizing antibodies to the 1F4 epitope in the Nicaraguan population, we observed different patterns of gain or loss of the neutralizing response attributable to this epitope between 3 and 18 months post-infection. A large subset of individuals retained the same proportion of the DENV1 type-specific response attributable to the 1F4 epitope at 3 and 18 months. Interestingly, a number of individuals gained DENV1 neutralization directed to the 1F4 epitope at the later time-point. In this subset, we observed a decay of overall DENV1 NT_50_ values and a slight decrease of DENV2 titers, which could be attributed to waning of cross-reactive titers^22,47^. Thus, in these individuals, 1F4-like antibodies accounted for a larger proportion of the DENV1 polyclonal neutralization at the later time-point. Conversely, a very small subset of individuals lost response to the 1F4 epitope at 18 months. Similar to our previous findings with the 5J7 epitope in primary DENV3 cases^31^, we observed a rise in the cross-neutralizing antibody titers in this small subset of individuals who lost recognition of the type-specific epitope. In all three groups, rDENV2/1 neutralizing antibody titers remained relative constant over time. The existence of multiple type-specific neutralizing epitopes within the DENV1 repertoire could explain the data showing decay of DENV1 neutralizing titers while the 1F4 neutralizing titers remain constant. Alternatively, the decay of DENV1 type-specific neutralizing activity may also be attributed to the increase in neutralizing titers to the heterologous serotypes. A number of recent studies in dengue-endemic areas have shown the maintenance of cross-reactive titers post-primary DENV infection, potentially due to “boosting” by heterotypic reinfection that fell short of the antibody threshold for a new infection^8,31,48^.

Numerous studies substantiate neutralizing antibody titers as correlates of protection for dengue disease^7–9^. Nonetheless, homotypic reinfections in Nicaragua^49^ and Peru^50^ have been reported, as well as breakthrough infections in individuals who seroconverted to the serotype in question following immunization^51,52^. One hypothesis to explain such observations is the genotypic variation within each serotype that may lead to neutralization escape. An increasing number of studies show the impact of genotypic variation on neutralizing responses^39,53–57^. Importantly, recent results from a DENV vaccine clinical trial pointed to a higher efficacy when the vaccine DENV4 genotype matched the one circulating in the area where immunization took place^39,51^. To compare the prevalence of the 1F4 epitope in areas affected by different DENV1 genotypes, we included samples from Sri Lanka, where genotype I circulates, in contrast with Nicaragua, where genotype V is found. Similar to the Nicaraguan population, the levels of 1F4 neutralization varied across the Sri Lankan individuals analyzed. However, a higher proportion of subjects from Sri Lanka had neutralizing antibodies that tracked with the DENV1 1F4 epitope compared to the Nicaraguan population. In the antigenic cartography analysis, the Sri Lankan DENV1 immune sera grouped closer to the rDENV2/1 strain compared to the Nicaraguan sera. The 1F4 epitope displayed in the rDENV2/1 chimera is derived from a DENV1 genotype IV strain. There are 2 amino acid residues that vary between the transplanted 1F4 epitope and the corresponding region on DENV1 genotype V (Nicaraguan strain) and 1 residue that varies between the transplanted 1F4 epitope and the corresponding region on DENV1 genotype I (Sri Lankan strain). Further studies are needed to determine if these specific changes or other factors are responsible for the observed differences in antibody specificity between Nicaraguan and Sri Lankan subjects. Taken together, our analyses highlight the importance of capturing intra-serotype genotypic variations when analyzing novel neutralizing epitopes, as well as the need for genotype-defined chimeric viruses. Following the findings showing that the Dengvaxia vaccine drives a DENV4 type-specific neutralizing antibodies response in many individuals^34^, sieve analysis indicated that vaccine efficacy was greater against DENV4 genotype II (vaccine-matched genotype) than genotype I^52^. Our studies herein may help with understanding the performance of vaccines that stimulate DENV1 type-specific neutralizing antibodies in DENV-seronegative children.

In sum, we demonstrate that the 1F4 epitope is an important component of the DENV1 type-specific epitope repertoire in a large number of individuals who experienced natural DENV1 infection. The differential recognition of this epitope across populations exposed to different DENV1 genotypes provides evidence that intra-serotype amino acid variations can lead to variation in neutralization of type-specific antibodies. Importantly, the substantial variation of 1F4 epitope recognition in both Nicaraguan and Sri Lankan populations suggest that additional epitopes within the DENV1 repertoire also drive type-specific neutralization and deserve further investigation.

## Material and Methods

### Ethics statement

The protocols for the Pediatric Dengue Cohort Study and the Pediatric Dengue Hospital-based Study in Nicaragua were reviewed and approved by the Institutional Review Boards of the University of California, Berkeley, (Cohort #2010-09-2245; Hospital #2010-06-1649) and the Nicaraguan Ministry of Health (Cohort NIC-MINSA/CNDR-CIRE-09/03/07-008.ver1; Hospital NIC-MINSA/CNDR-CIRE-01/10/06-13.Ver 14). Parents or legal guardian of the subjects enrolled in these studies provided written informed consent, and participants 6 years of age and older provided assent. Ethical approval for this research was obtained from the Ethical Review Committee of the Faculty of Medicine, University of Colombo, Sri Lanka. The University of North Carolina (UNC) institutional review board determined that its approval was not required because participating UNC investigators were not involved in human subject research (Exemption #14-0195). Only subjects who provided written informed consent were enrolled in the study.

### Study population

(i) Study enrollment took place at Hospital Infantil Manuel de Jesús Rivera, the Nicaraguan national pediatric reference hospital. Children ages between 6 months and 14 years suspected of DENV infection (<7 days since onset of symptoms) were eligible to participate in the hospital study, as described previously^58^. Laboratory-confirmed cases were classified by disease severity according to the 1997 WHO guidelines^4^ using a computerized algorithm that compiled all clinical data meeting all criterion for dengue fever (DF), dengue hemorrhagic fever (DHF), or dengue shock syndrome (DSS)^58^. Plasma samples were collected in the acute (days 1 to 6 of illness) and convalescent (days 14 to 28 post-onset of symptoms) phases, as well as 3, 6, 12, and 18 months after illness. (ii) The Pediatric Dengue Cohort Study is an ongoing prospective dengue cohort study that follows approximately 3,700 children ages 2–14 in District II of Managua, Nicaragua^59^. Healthy annual blood samples collected from 4 participants from year 1 through 4 post-primary DENV1 infection were used. (iii) In Sri Lanka, convalescent DENV-immune sera were obtained from individuals participating in a hospital-based febrile illness study that recruited subjects suspected of dengue (samples collected 20–65 days after laboratory confirmed DENV1 infection), which is fully described in Raut *et al*.^60^ or from healthy individuals who donated blood to a blood bank and had monotypic neutralizing antibody to DENV1 only.

### Laboratory tests

In the Nicaraguan studies, DENV infection was identified by type-specific RT-PCR for detection of viral RNA^61^, isolation of DENV on C6/36 cells^61^, and/or seroconversion by IgM enzyme-linked immunosorbent assay (ELISA)^62^ or a > 4-fold increase in total antibody titer as measured by inhibition ELISA in paired acute- and convalescent-phase samples^10,63^. In the hospital study, primary dengue cases were determined by inhibition ELISA, where antibody titers of <2,560 in days 14–28 post-onset of symptoms (early convalescent phase) defined primary infection status^58^. In the cohort study, primary infection with DENV was detected by seroconversion (a titer of <1:10 to >1:10 as determined by Inhibition ELISA) in paired consecutive annual samples^59^. Inhibition ELISA on paired healthy annual samples demonstrate that after the first infection with DENV1, no >4-fold increase in antibody titers is seen (Supplementary Table S2), indicating that these individuals did not experience subsequent infection from year 1 through year 4 post-primary infection. This is supported by the observation of no increase in NT_50_ titers over the 4-year period (Fig. 5A,B). In the Sri Lankan studies, primary DENV1 infections were defined as individuals with fever whose acute specimen tested positive for DENV1 by PCR and negative for DENV-specific IgG^64,65^. From seven individuals with laboratory-confirmed primary DENV1 infections, convalescent specimens collected 20–65 days after presentation were used for the current study. We also identified people with primary DENV1 immunity by screening healthy individuals in Sri Lanka for the presence of neutralizing antibodies to the 4 DENV serotypes as previously described^66^. Five specimens from individuals with neutralizing antibodies to DENV1 only (monotypic) were also used for the current study.

### Cells and viruses

U937 cells expressing DC-SIGN (dendritic cell-specific intracellular adhesion molecule-3-grabbing nonintegrin), a known DENV attachment factor, were used for the neutralization assays. The U937 cells were maintained as suspension cell cultures at 37 °C with 5% Co_2_ in RPMI 1640 (Gibco) supplemented with 1% non-essential amino acids, 1% penicillin and streptomycin, and 5% fetal bovine serum (FBS, HyClone). Propagation of the parental DENV1 (West Pac 74) and DENV2 (S16803) viruses and recombinant virus rDENV2/1 was performed in *Aedes albopictus* C6/36 cells grown at 32 **°**C in 5% Co_2_.

### DENV neutralization assay

To measure DENV-specific neutralizing antibodies, we employed a flow cytometry-based assay, as previously described^67^. Briefly, DENV-immune plasma samples at an initial dilution of 1:5 were serially diluted 3-fold 8 times in RPMI supplemented with 2% FBS. A dilution of virus that infects between 8–15% of the U937 cells (previously determined by virus titration) was added to the plasma dilutions and incubated for 1 h at 37 **°**C. Infection was carried out in a 96-well plate by mixing, in each well, 20 uL of virus with 50,000 U937DC-SIGN cells in a total volume of 100 µl complete RPMI media. The cells were then incubated at 37 °C in 5% CO_2_ for 24 hours. Next, cells were fixed in 4% paraformaldehyde, incubated for 10 min at room temperature (RT), and centrifuged at 252 × g for 5 min. Subsequently, cells were blocked in permeabilization buffer (0.1% saponin, 5% bovine serum albumin in 1X phosphate-buffered saline [PBS]) for 30 min at RT. Then, cells were incubated with anti-E mAb 4G2 conjugated to Alexa 488, diluted in blocking buffer (0.5% bovine serum albumin and 0.02% sodium azide in 1X PBS) for 25 min at RT. Finally, cells were washed and resuspended in PBS. Acquisition of the infected cells was performed with a Guava flow cytometer (EMD Milipore) by gating Alexa 488-positive cells. The neutralizing antibody titer that reduced the infection by 50% (NT_50_) was calculated by a nonlinear, 4-parameter dose-response regression analysis with Prism software (GraphPad), which is expressed as the reciprocal serum dilution. Data generated had to fit the quality control criteria, where the sigmoidal dose-response regression fit included an absolute sum of squares of <0.2 and a coefficient of determination (R^2^) of >0.9.

### Statistical analysis

Statistical analysis was performed using Prism Graph Pad 5.0 (La Jolla, CA). One-way analysis of variance (ANOVA) was used to compare the NT_50_ values to the chimeric virus and parental viruses at early convalescent, 3 and 18 months post-illness. Paired t test was used to compare the proportions of the DENV1 type-specific neutralizing response attributable to the 1F4 epitope between samples collected between 3 and 18 months post-infection. Statistical difference was considered significant when p-value was <0.05.

## Supplementary information

Supplementary Info #
