# Supplementary material for: Tracking the polyclonal neutralizing antibody response to a dengue virus serotype 1 type-specific epitope across two populations in Asia and the Americas
Source: Sci Rep. 2019 Nov 7;9:16258. doi: 10.1038/s41598-019-52511-z (PMC6838341; doi:10.1038/s41598-019-52511-z)
Supplement: Supplementary file 1 — Supplementary Info # [file 41598_2019_52511_MOESM1_ESM.pdf]

SUPPLEMENTARY INFORMATION

Tracking the polyclonal neutralizing antibody response to a dengue virus serotype 1 type-specific epitope across two populations in Asia and the Americas

Daniela Andrade, Colin Warnes, Ellen Young, Leah C. Katzelnick, Angel Balmaseda, Aravinda M. de Silva, Ralph S. Baric, and Eva Harris

Dengue Virus Strains	Residue changes in 1F4 within envelope protein (EDI and EDII)																										
	**46**	**47**	**49**	**50**	**51**	**52**	**136**	**138**	**155**	**156**	**157**	**160**	**161**	**162**	**163**	**165**	**168**	**170**	**171**	**172**	**173**	**174**	**176**	**177**	**273**	**274**	**275**	**276**
Nicaragua DENV1 – Genotype V	L	L	T	E	V	T	K	S	T	T	E	T	I	A	T	T	A	T	S	E	I	Q	T	D	S	G	T	T
Sri Lanka DENV1 – Genotype I	L	L	T	E	V	T	K	S	S	T	E	T	T	A	T	T	A	T	T	E	I	Q	T	D	S	G	T	T
West Pacific DENV1 – Genotype IV	L	L	T	E	V	T	K	S	T	T	E	T	T	A	T	T	A	T	S	E	I	Q	T	D	S	G	T	T
Thailand DENV2	L	I	T	E	A	K	E	T	T	G	K	K	E	I	K	T	S	I	T	E	A	E	T	G	S	S	G	N

Supplementary Figure S1. Amino acid alignment between parental DENV2 and parental DENV1 genotypes V, I and IV. The amino acid alignment of Nicaraguan DENV1 (genotype V), Sri Lankan DENV1 (genotype I) and West Pac DENV1 (genotype IV) reveals residue substitutions in the 1F4 epitope (highlighted in pink), in contrast with the DENV2 backbone (highlighted in yellow).

Supplementary Table S1. Characteristics of the 20 individuals enrolled in the Nicaraguan hospital-based study.

Code	Age	Gender	Disease severity	Year of infection	PCR-confirmed serotype
1449	13	female	DF	2012	DENV1
1507	13	male	DF	2012	DENV1
1500	2	male	DF	2012	DENV1
1415	12	male	DF	2012	DENV1
1447	9	female	DF	2012	DENV1
1495	10	male	DF	2012	DENV1
1491	6	male	DF	2012	DENV1
1487	14	male	DHF	2012	DENV1
1385	12	male	DF	2012	DENV1
1366	4	female	DF	2012	DENV1
1458	13	female	DF	2012	DENV1
1397	8	female	DF	2012	DENV1
256	9	female	DF	2007	DENV1
312	4	male	DF	2007	DENV1
1385	12	male	DF	2012	DENV1
1338	7	male	DHF	2012	DENV1
1342	6	male	DF	2012	DENV1
1379	5	male	DF	2012	DENV1
1454	6	female	DHF	2012	DENV1
1408	6	male	DF	2012	DENV1

Supplementary Table S2. Characteristics of the four individuals enrolled in the Nicaraguan cohort study.

Code	Age	Gender	Disease severity	Year of infection	PCR-confirmed serotype	iELISA titers				
						**year 0**	**year 1**	**year 2**	**year 3**	**year 4**
3675	10	female	DF	2009	DENV1	2	341	323	236	N/A
						**year 0**	**year 1**	**year 2**	**year 3**	**year 4**
1396	9	female	DF	2005	DENV1	2	103	N/A	394	333
						**year 0**	**year 1**	**year 2**	**year 3**	**year 4**
2126	6	female	DF	2005	DENV1	2	516	N/A	321	331
						**year 0**	**year 1**	**year 2**	**year 3**	**year 4**
2934	6	male	DF	2005	DENV1	2	1171	N/A	621	400
